# Immune regulation of neurodevelopment at the mother–foetus interface: the case of autism

**DOI:** 10.1002/cti2.1211

**Published:** 2020-11-13

**Authors:** Stefano Sotgiu, Salvatorica Manca, Antonella Gagliano, Alessandra Minutolo, Maria Clotilde Melis, Giulia Pisuttu, Chiara Scoppola, Elisabetta Bolognesi, Mario Clerici, Franca Rosa Guerini, Alessandra Carta

**Affiliations:** ^1^ Unit of Child Neuropsychiatry Department of Medical Surgical and Experimental Sciences University of Sassari Sassari Italy; ^2^ Unità Operativa di Neuropsichiatria Infanzia e Adolescenza (UONPIA) ASSL Sassari Sassari Italy; ^3^ Child & Adolescent Neuropsychiatry Unit Department of Biomedical Sciences University of Cagliari Cagliari Italy; ^4^ IRCCS Fondazione Don Carlo Gnocchi ‐ ONLUS Milan Italy; ^5^ Department of Pathophysiology and Transplantation University of Milano Milan Italy

**Keywords:** autism spectrum disorder, maternal immune activation, inflammation, autoimmunity, gestation, neurodevelopment

## Abstract

Autism spectrum disorder (ASD) is a neurodevelopmental disorder defined by deficits in social communication and stereotypical behaviours. ASD’s aetiology remains mostly unclear, because of a complex interaction between genetic and environmental factors. Recently, a strong consensus has developed around ASD’s immune‐mediated pathophysiology, which is the subject of this review. For many years, neuroimmunological studies tried to understand ASD as a prototypical antibody‐ or cell‐mediated disease. Other findings indicated the importance of autoimmune mechanisms such as familial and individual autoimmunity, adaptive immune abnormalities and the influence of infections during gestation. However, recent studies have challenged the idea that autism may be a classical autoimmune disease. Modern neurodevelopmental immunology shows the double‐edged nature of many immune effectors, which can be either beneficial or detrimental depending on tissue homeostasis, stressors, neurodevelopmental stage, inherited and *de novo* gene mutations and other variables. Nowadays, mother–child interactions in the prenatal environment appear to be crucial for the occurrence of ASD. Studies of animal maternal–foetal immune interaction are being fruitfully carried out using different combinations of type and timing of infection, of maternal immune response and foetal vulnerability and of resilience factors to hostile events. The derailed neuroimmune crosstalk through the placenta initiates and maintains a chronic foetal neuroglial activation, eventually causing the alteration of neurogenesis, migration, synapse formation and pruning. The importance of pregnancy can also allow early immune interventions, which can significantly reduce the increasing risk of ASD and its heavy social burden.

## Graphical abstract

In this review, we argue over the significance of the several immune signatures associated with both susceptibility to autistic disorder and susceptibility to its core social and behavioural traits. Despite conclusive evidence, an impaired mother–child immune interplay during pregnancy is thought to be aetiopathogenetically crucial for the atypical neurodevelopment as observed in autism, and an appropriate management of immune responses during pregnancy may positively impact on autism.

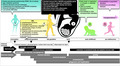

## Introduction

In the last few decades, a strong consensus has developed around the immune aetiology of autism spectrum disorder (ASD), particularly after the description of a chronic microglial activation in brains of individuals with ASD.^1^ From then on, an increasing amount of studies have described the association and causative links between ASD and several aberrant immune functions. In this review, we summarise some of the results accumulated in the last 20 years and discuss whether the (auto)immune theorisation on ASD is a solid argument, a self‐perpetuating tautology or a still undetermined issue.

## Autism spectrum disorder

Autism is a multifaceted neuropsychiatric disorder defined by a constellation of early‐appearing social communication deficits and repetitive sensory‐motor behaviours, with a strong male preponderance.^2^ ASD frequency has progressively increased in the last decades up to the current rate of 1 of 59 children in the USA.^3^ This escalation is generating a substantial burden on public health care^2^, ^3^ and is only partly attributable to a higher public awareness and better diagnostic criteria. Yet, the majority of autistic disorders still remains of undetermined origin.^4^ ASD causation and clinical pleiotropy involve a complex combination of interacting genetic, epigenetic and immunological factors, which in isolation are non‐causative.^2^, ^4^, ^5^, ^6^

The core autistic features, its onset and clinical trajectories may vary substantially, allowing clinicians to define distinct ASD subtypes.^2^ Many individuals present with cognitive or psychiatric comorbidities and minor neurological or morphological atypia (e.g. microcephaly). This subset, termed 'complex autism', is phenotypically distinguishable from the non‐dysmorphic and less‐comorbid 'essential autism'.^7^ Individuals with 'complex' ASD are more likely to carry pathogenic *de novo* mutations and copy‐number variants.^8^ Also heterogeneous is the timing of clinical appearance, according to which ASD is categorised into 'early' or 'regressive' patterns of onset. In the early‐onset type, children display early atypical behaviour and social communication delay; in the regressive pattern, loss of communication is experienced later, conventionally after the child has learned 5 words and used them for at least 3 months.^9^ What substantially distinguishes 'complex' versus 'essential' or 'early' versus 'regressive' ASD forms is yet to be uncovered. It is believed that most children with early‐onset ASD have more frequently a 'complex' pattern, with lower intelligence quotient and a male/female ratio close to 1, while the regressive onset is prevalent in the 'essential' subtype and in male children.^7^, ^8^, ^9^

Interestingly, infants 'at risk' of ASD (i.e. with an autistic sib) and later diagnosed with autism have a transient excess volume of extra‐axial cerebrospinal fluid (CSF) confirmed using brain magnetic resonance imaging (MRI), in which ASD precedes the onset of behavioural symptoms (between 6 and 24 months of age) and does not correlate with reduced parenchymal size, which, in fact, is increased in most cases.^10^

MRI studies also show that abnormal brain enlargement is more common in male children with regressive ASD onset, while brain size in boys without regression does not differ from controls.^11^ Retrospectively, head circumference in boys with regressive autism was normal at birth and diverged at about 4–6 months.^11^ The rapid, although transient, head growth in the regressive subtype is associated not only with known genes such as phosphatase and tensin homolog (PTEN) and chromodomain helicase DNA‐binding protein 8,^4^ but also with *de novo* mutations or other candidate genes or immunological dysfunctions.^12^ These findings suggest a strategy of phenotype–genotype–immunotype combination to further elucidate the complex relationships leading to ASD clinical heterogeneity.

## On the immune‐mediated origin of ASD

### Familial autoimmunity and poly‐autoimmunity in ASD patients

Families with at least one autistic child tend to display a high autoimmune burden such as type 1 diabetes, thyroiditis and maternal rheumatoid arthritis (RA). The risk of ASD in offspring is particularly increased when maternal autoimmunity is on an active phase during pregnancy,^13^ suggesting that an active inflammatory state during gestation may negatively influence the foetal neurodevelopmental trajectory.

Individuals with ASD very often manifest allergic and autoimmune comorbidities early in life or during adolescence. Type 1 diabetes, asthma, allergic rhinitis, atopic dermatitis and gastrointestinal problems, including Crohn’s and coeliac diseases, are over‐represented and, in some individuals with ASD, may even influence their behaviour and the severity of core clinical features.^14^ Notably, gene expression of blood–brain barrier and intestinal tight junction proteins are found to be similarly affected on *post‐mortem* tissues of subjects with ASD, which raised the hypothesis, termed the 'gut–brain axis',^15^ that microbiota and intestinal metabolites could directly influence the brain when the blood–brain barrier is not sufficiently intact.

### Gestational infections and foetal neurodevelopment

The placenta is a selective barrier that enables nutrient absorption and waste elimination, provides protection from pathogens and allows defensive maternal immunoglobulins to positively cross into the amniotic fluid compartment. Overall, a healthy pregnancy requires a fine balance of the maternal immune activation to maintain a protective, homeostatic, non‐inflammatory environment, to ensure a tolerance state and to avoid rejection of the semi‐allogeneic foetal–placental unit.^16^

However, as the developing foetal brain goes into an expedited neuroplasticity process (i.e. neurogenesis, neuronal differentiation, migration, synapse formation and apoptosis), it is particularly vulnerable to intrauterine (e.g. maternal undernutrition or diabetes) and, perhaps, early postnatal adversities.^17^ It is increasingly recognised that even minor perturbations of the gestational immune environment (e.g. common infections or a mere cytokine imbalance) may have deleterious consequences on early neurodevelopment.^17^, ^18^

The vast majority of women experience at least one infection during gestation, and thus, most foetuses undergo a modification in their immune environment. Unfortunately, in the least resilient foetuses the infection may alter the growing trajectory, an alteration that, combined with other genetic and environmental susceptibility factors, may eventually result in an ASD diagnosis during early years.^18^

Many studies of prenatal exposure to rubella and cytomegalovirus have linked maternal infections with neurological, psychiatric, cognitive and mood disorders in descendants, the strongest association being with schizophrenia and ASD.^19^ A large Danish study reported a threefold increase in risk of ASD in the offspring of pregnant mothers with viral infection in the first trimester or bacterial infection in the second trimester, suggesting the importance of both the infectious agent itself and the foetal maturation stage.^20^ Regardless of the type of infection (viral, bacterial, severe or moderate) and regardless of pregnancy stage, febrile episodes longer than one week have been associated with a high risk of ASD in offsprings.^21^

**Figure 1:**
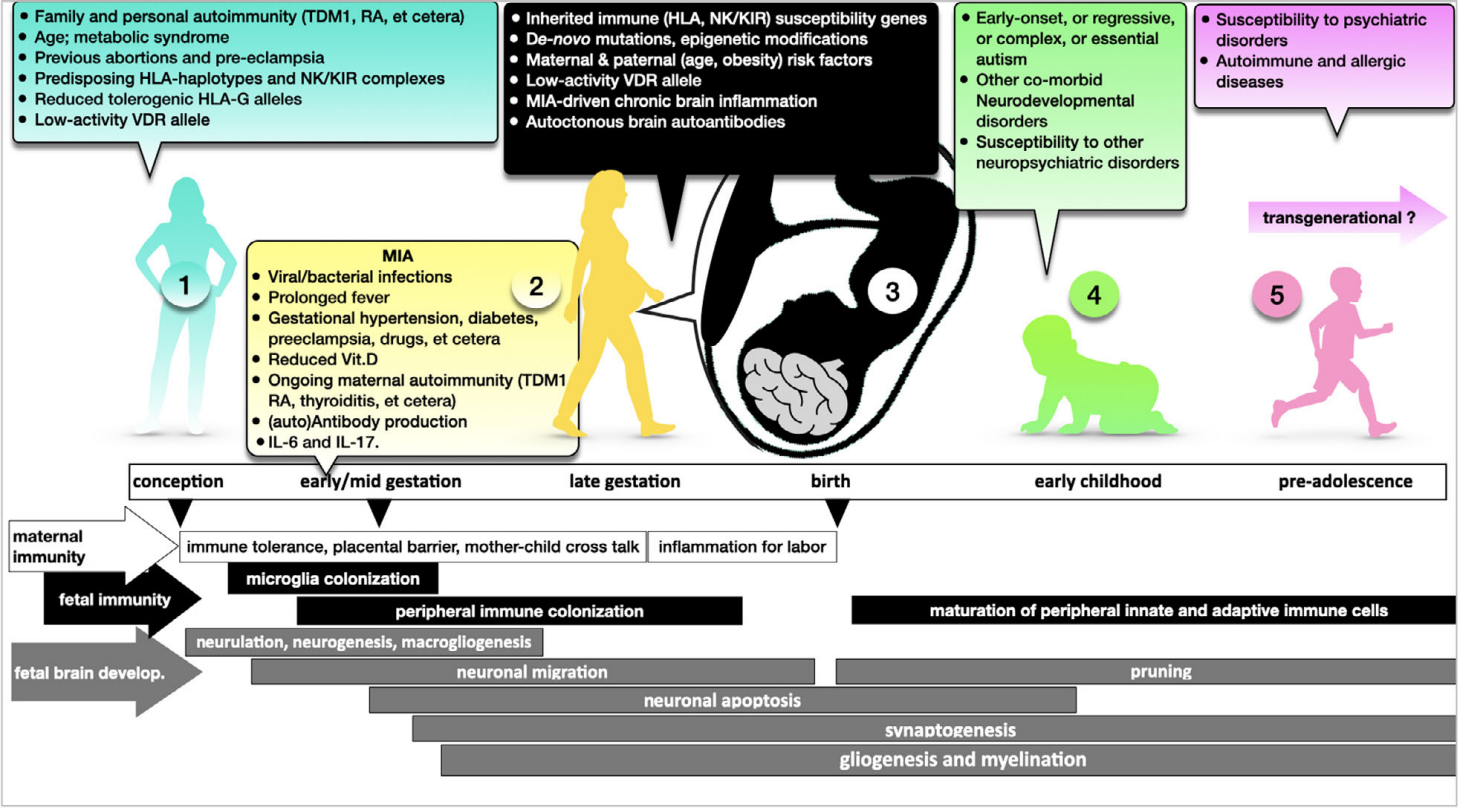
Multistep immunological road to autism is as follows: the first step (1) starts from the maternal immunogenetic predisposition to MIA (KIR/HLA), familial and personal autoimmunity and epigenetic forces such as age and gestational metabolic syndrome; the second step (2) takes place at the intrauterine level where pathogens and other events can trigger a detrimental immune activation at the maternal–foetal interface; and the third crucial step (3) takes place at the brain foetal level, at a crossroad between foetal immune and non‐immune genetic susceptibility, chronic microglial activation and persistent maternal immune activation. As a result of the complex interaction of different risk factors and depending on several variables, (3) and (4), offspring may present with various clinical forms of ASD (early onset, complex, regressive and essential). The same immune and structural brain alterations may predispose a toddler, or even an adolescent (5), to a late onset of neuropsychiatric illnesses. The transgenerational effect of the previous multistep process is, in humans, only hypothesised. TDM1 = type 1 diabetes mellitus; RA = rheumatoid arthritis; NK/KIR = NK/killer cell immunoglobulin‐like receptor; VDR = vitamin D receptor; MIA = maternal immune activation; Vit.D = vitamin D; IL = interleukin.

From a mechanistic perspective derived from studies on human amniotic fluid^22^ and particularly from animal models,^23^ infection‐driven maternal serum cytokines, such as interleukin (IL)‐6 and IL‐17, can cross the placenta and stimulate the downstream production of other detrimental immune mediators of brain damage in the human foetal compartment, thus inducing behavioural and cognitive characteristics, strictly resembling human ASD features.^24^, ^25^, ^26^ Most studies agree on the concept that, depending on the embryonic/foetal developmental stage, the more severe the infection, the stronger the inflammatory response (and the fever), and the higher the risk of ASD in the developing child (Figure 1).^27^

### Maternal immune activation (MIA) and mother–child immune crosstalk

The maternal immune activation (MIA) during pregnancy in mammals, including humans, is being used as an experimental procedure to explore the phenomenology underlying risk and/or resilience of the foetus to neurodevelopmental disorders under gestational infectious/inflammatory circumstances.^23^, ^24^, ^25^, ^26^, ^27^, ^28^ MIA in experimental animals is classically modelled by injecting viral‐like or bacterial‐like molecules in pregnant rodents. Progeny displays both structural brain modification and behavioural anomalies explicitly evocative of the human autistic disorder, which can persist into adulthood.^29^, ^30^

In MIA‐induced ASD‐like behaviours, a combination of maternal chemokines and cytokines (e.g. IL‐6, IL‐17, IL‐4)^29^, ^31^ crosses the placenta, acts on the developing foetal brain either directly or by altering its epigenetic regulation, and leads to detrimental actions on its plasticity, precursor migration and synaptic pruning.^32^ About 10% of mothers of children with autism has been found to produce anti‐foetal brain antibodies.^33^ These autoantibodies can induce ASD‐like pathology and ASD‐like behaviour in animal models^33^ and target a combination of foetal brain antigens such as lactate dehydrogenase‐A/B, stress‐induced phosphoprotein‐1, collapsin response mediator protein 1/2 and Y‐box binding protein, acting by reducing the dendritic spine formation in the mouse cortex.^34^

Studies of experimental models of MIA now take into account different infection types (e.g. viral versus bacterial, acute versus chronic), different gestational periods in which exposure occurs, additional maternal stressors such as hypernutrition and starvation^35^ or known human maternal risk factors for neurodevelopmental disorders such as ageing or gestational metabolic syndromes.^5^, ^6^ In mice, offspring response to hostile postnatal events, such as maternal care by a surrogate mother, and gender‐specific factors are being evaluated.^36^ When impacted by prenatal immune disruption, female offspring exhibit a less severe neurodevelopmental disorder than male offspring, which may be explained by a sex‐driven modulation of several immune genes in response to metabolic and inflammatory stress.^36^ Thus, the MIA model is evolving into a translational model for human research on transgenerational transmission of atypical neurodevelopment^37^ and on mental health at large.^31^, ^35^ As the vast majority of pregnant women exposed to infections give birth to neurotypical offspring, understanding which pregnancies are fragile and which are resilient could substantially reduce the risk of ASD, attention‐deficit/hyperactivity disorder (ADHD), intellectual disability, and other neurodevelopmental disorders and common human psychiatric disorders such as schizophrenia and bipolar disorders (Figure 1).

### Microglia and innate immunity

Microglia are innate immune cells that colonise the brain in the early pregnancy. They have a crucial role in the correct neurodevelopment of the child: they modulate astrocytic differentiation from neuronal precursor cells and, later, contribute to synaptic pruning and clearance of apoptotic neuronal precursors.^38^, ^39^, ^40^ Thus, a cyclic activation of microglia is positively required for a typical neurodevelopment to occur; on the contrary, persistent microglial activation causes brain cell death and reduced or abnormal interneuronal connectivity.^41^, ^42^

The 2005 paper by Vargas and coll.^1^ might represent the foundational moment in the theorisation of the inflammatory causes of ASD.^43^ Here, for the first time, a chronic activation process of microglial and astroglial cells was documented in several cortical regions of autoptic brains of subjects with ASD. Interestingly, the loss of Purkinje neurons complemented the presence of anti‐inflammatory cytokines and astroglia, suggesting a compensatory immune‐modulating process aimed at reducing the chronic inflammation of the damaged tissue.^1^, ^44^

Many studies confirmed these seminal findings and added the information that ASD pathology is neither age‐related nor colocalised with markers of acute inflammation, confirming a long‐standing rather than acute immune brain alteration.^45^ Furthermore, pro‐inflammatory cytokines were found elevated within the CSF and many brain regions, and the presence of CD68^+^ perivascular cells suggested a peripheral macrophage/monocyte brain infiltration through the blood–brain barrier.^46^

**Figure 2:**
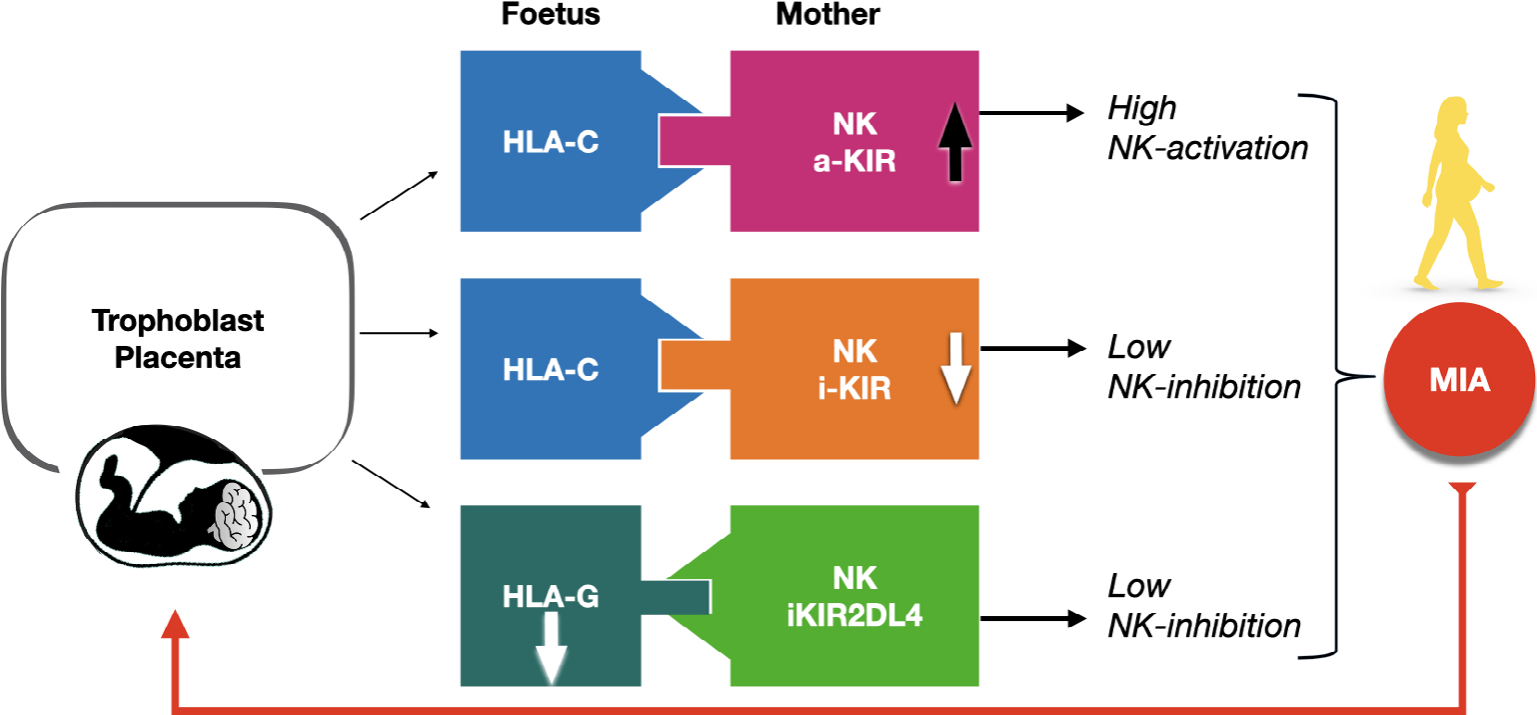
KIR‐HLA‐C and KIR/HLA‐G complexes are skewed in ASD children and their mothers because of the significant increase in NK cell pro‐activatory complexes and reduction in the tolerogenic ones. The net result causes NK‐activating molecules to prevail.^63^ We also found that cognitive and behavioural impairments are correlated with particular pro‐inflammatory KIRs‐HLA‐C complexes/HLA‐G*14bp + polymorphisms.^65^ In principle, the resulting NK activation negatively influences the development and the plasticity of the foetal central nervous system. NK = innate natural killer cells; a‐KIR = NK‐activatory killer cell immunoglobulin‐like receptor; iKIR = NK‐inhibitory killer cell immunoglobulin‐like receptor.

Subsequent transcriptomic analyses of autoptic brains from autistic subjects confirmed a strong upregulation of the brain’s innate immune pathways.^47^, ^48^, ^49^ Other analyses showed a dysregulated complement‐mediated synaptic pruning in brains from patients with ASD, presumably through microglia, and emphasised that the upregulation of interferon response and gene products of microglia and astrocytes is peaking during early development and coincident with clinical onset (Figure 2).^50^ In contrast, in schizophrenia and bipolar disorders, the microglial transcriptomic module appeared to be downregulated.^50^, ^51^

The presence of a chronic microglial activation in brains of individuals with ASD has been also substantiated *in vivo* by brain positron emission tomography using specific microglial radiotracers, which showed a significant activated microglial binding in cerebellum, in corpus callosum and in other cortical regions.^52^ Hypomethylation of some immune genes causing over‐transcription of TNF‐α, complement and microglial‐derived factors has also been found in several cortical areas of brains of individuals with ASD.^53^

### Genes, immunity and autism

Inherited factors have an undeniable role in both the susceptibility to and the clinical complexity of ASD,^4^, ^8^ as shown by the higher concordance rate among monozygotic compared with dizygotic twins. However, inherited genes may account for only 20% of ASD cases, as they are also present in individuals with typical neurodevelopment.^4^, ^54^ Since the concordance in dizygotic twins is higher than that of non‐twin siblings and is progressively increasing, other drivers are necessarily operating during gestation.^6^ Among these, d*e novo* mutations are emerging as a new key factor, and are enriching the genome of many individuals with neurodevelopmental disorders, in particular, ASD.^54^

Discussions of ASD‐associated or putatively causative immune genes are extraordinarily abundant in literature. An early dispute was about the involvement of the human leucocyte antigen (HLA) in ASD, since different associations were reported in different studies. Class‐I HLA‐B44 and HLA‐B15, HLA‐DRB1*04 (the major susceptibility allele for rheumatoid arthritis) and other class II alleles sharing the third hypervariable region were associated with ASD in various studies among Caucasians,^55^, ^56^ with DRB1*13 having some protective functions.^55^ HLA‐DRB1*03‐DQB1*02 and DRB*07‐DQB1*08 haplotypes of individuals with ASD were associated with high IgA response to tissue transglutaminase II, an enzyme involved in the immune‐mediated pathophysiology of the coeliac disease,^57^ confirming a link between HLA and autoimmune ASD comorbidities. Our studies in families with at least one affected child suggested significant associations with the α and β blocks, conserved HLA regions mapping classical HLA‐A, HLA‐B and non‐classical HLA‐G alleles.^58^, ^59^

### Immune genes and cells at the maternal–foetal interface

The mother–child interface allows the foetus to safely develop in the uterus, in spite of being identified as half‐allogenic by the maternal immune system. A large variety of decidual leucocytes are crucial in this control, including innate natural killer (NK) and lymphoid cells, dendritic cells, NKT cells, and regulatory T and B cells (Tregs and Bregs). Of particular interest is an aberrant leucocyte balance in the placenta, which is significantly associated with pregnancy loss and infertility.^60^

Innate CD56^+^CD3^‐^ NK cells are possibly the largest cell subpopulation at the maternal–foetal interface during early pregnancy, with several properties, including immunosurveillance and promotion of foetal growth.^61^ By targeting activated immune cells, virally infected cells and also placental cells through an interaction between their killer cell immunoglobulin‐like receptors (KIRs) and the cognate HLA ligands, NK cells play a crucial role in the protection of the foetus.^62^

However, when conditioned by a particular KIR‐HLA ligand, activated NK cells can produce pro‐inflammatory cytokines, perforin and granzyme B, and induce detrimental innate immune responses.

Torres and coll. first described an increased frequency of KIR‐activating genes and their HLA ligands (HLA‐C and HLA‐G, mapping in the α and β blocks) in autistic children.^55^ We confirmed that pro‐inflammatory KIR/HLA are higher, whereas tolerogenic KIR/HLA gene complexes are lower in children with ASD and, more significantly, in their mothers.^63^ This suggests that the true HLA‐ASD association should be viewed as a dysregulated interaction between maternal KIRs and filial HLA (Figure 2). We also evaluated HLA‐G allelic frequency in families of autistic children and found that the tolerogenic HLA‐G*01:01 allele is less common, whereas the NK‐activating HLA‐G*01:05N allele is more frequent in affected children and their mothers than controls (Figure 2),^64^ and correlated with the extent of behavioural disorder in autistic children.^65^ Also, mothers of children with ASD show a HLA‐G allele distribution similar to that of women with recurrent miscarriages, reinforcing the idea that an intrauterine pro‐inflammatory milieu may threaten a typical neurodevelopment or, at worst, cause miscarriage.^64^

### Peripheral innate and adaptive immunity

Compared with control children, markers of a dysregulated innate immunity appear in autism also at the peripheral level with increased number of NK cells, increased production of perforin and granzyme B,^66^ and upregulated expression of KIR, IL‐1RA and HLA‐DR, which correlates with regressive ASD features and bigger amygdala size.^12^, ^43^, ^67^ IL‐1β, IL‐6, IL‐8, interferon‐γ and monocyte chemoattractant protein‐1 are also abnormally elevated, with a significant reduction in the concentration of the anti‐inflammatory transforming growth factor‐β1.^68^ A transcriptomic analysis of blood samples showed that, overall, differences in gene expression between sibling pairs discordant for ASD mainly reflect changes in NK cell subpopulation.^69^

**Figure 3:**
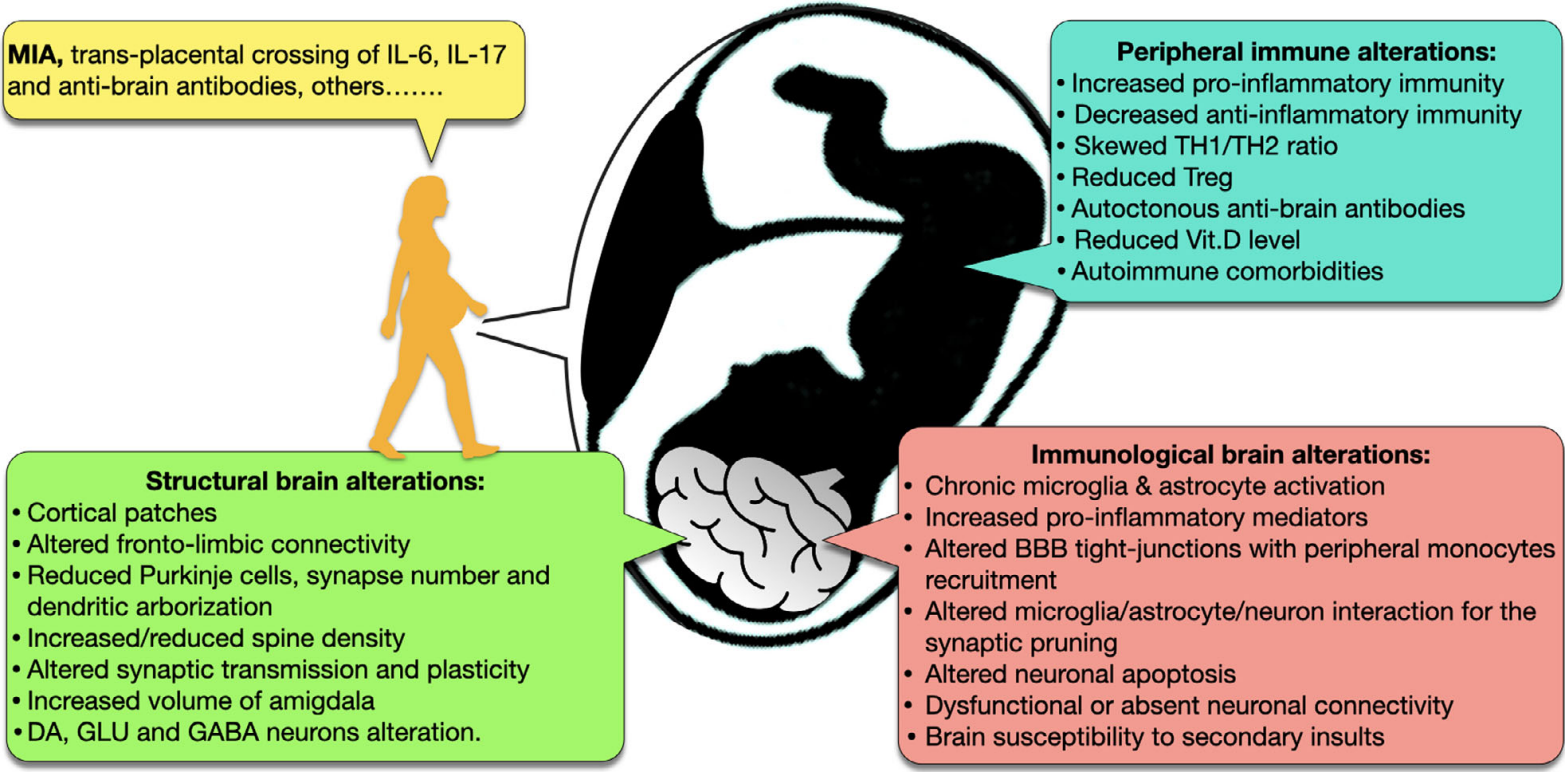
Main structural and immunological brain alterations in autism are linked to an abnormal maternal immune activation (MIA) and, in humans, are also possibly linked to the transdecidual crossing of pro‐inflammatory cytokines and autoantibodies against foetal brain antigens. Peripheral immune alterations of the foetus are mainly of the innate type. IL = interleukin; DA = dopaminergic; GLU = glutamatergic; GABA = γ‐aminobutyric acid; NK/KIR = NK/killer cell immunoglobulin‐like receptor; TH = T helper cells; Treg = T regulatory cells; Vit.D = vitamin D; BBB = blood–brain barrier.

In contrast to the innate counterpart, an alteration of the adaptive immunity in the peripheral blood is perhaps marginal in ASD phenomenology. Serum anti‐cardiolipin, anti‐2‐glycoprotein, anti‐M1 ganglioside, anti‐endothelial cells, anti‐double‐stranded DNA and anti‐mitochondrial DNA autoantibodies have been observed in individuals with ASD along with antibodies targeting brain self‐antigens.^43^, ^70^ However, as they are also detected in typically developing controls, their role may be merely epiphenomenal rather than causative.^71^ Nevertheless, compared with no reactivity in controls, 20% of autistic children have serum immunoreactivity to GABAergic neurons of the primate cerebellum, which correlates with a worse score on their Child Behavior Checklist,^72^ cognitive impairment and motor stereotypies (Figure 3).^73^

As for the T‐cell compartment, newborns who were later diagnosed with ASD show early immune dysregulation such as increased monocyte chemoattractant protein‐1, IL‐4 and IL‐1β.^74^ Later in life, autistic children present with other dysregulated T‐cell activities, including the excess production of IL‐5, IL‐12, IL‐13, IL‐17, IL‐21, IL‐22 and IL‐23, and a skewed CD4/CD8 ratio associated with decreased executive function.^12^, ^14^, ^75^ Clustering subjects into TH1 and TH2 endophenotypes revealed that the TH1 profile correlates with a more severe ASD behavioural phenotype.^76^ A reduced apoptotic signal (Fas) receptor CD95 together with increased HLA‐DR and CD26 suggests a persistent peripheral T‐cell activation in ASD.^12^ Other studies found circulating CD4^+^ CD25 high Tregs to be functionally impaired particularly in children with clinically severe form of ASD (Figure 3).^77^

However, recent large biomarker profilings in discordant sibling pairs do not confirm this adaptive immune dysregulation nor its association with ASD clinical severity. Only one correlation was found between the level of the innate cytokines IL‐1α/β and quantitative traits at the Vineland Adaptive Behavior Scale.^78^, ^79^

## Towards an immunological treatment of ASD

### Prenatal translational studies

On an experimental level, serum IL‐6 and IL‐17 elevations in MIA dams are necessary and sufficient to induce brain and behavioural alterations in offspring, since their treatment with specific antibodies prevents behavioural abnormalities in the progeny.^23^, ^80^ The translational value of these studies has been supported by *in vitro* ^22^ and structural and functional MRI studies in humans.^22^, ^24^, ^25^, ^26^, ^30^

Similar experimental results, with a high translational value, are achieved with non‐classical immunological treatments, such as dietary interventions. The (omega‐3) docosahexaenoic acid (DHA) is important for foetal development, and neuronal and immune functions as it reduces detrimental IL‐6 production. In humans, low maternal DHA during gestation has been associated with cognitive and behavioural abnormalities.^81^ In animals, comparing dams assigned to a DHA‐deficient diet with those with a DHA‐enriched diet showed that DHA‐enriched animals have offspring with less behavioural problems compared with the DHA‐deficient counterpart.^82^ Likewise, memory impairment of MIA model offspring was restored by zinc supplementation during pregnancy,^83^ although the translational relevance of this experiment for therapeutic interventions in humans is low. Pre‐treatment of vitamin D (Vit.D; 25‐hydroxycholecalciferol) during experimental MIA induction prevents stereotyped digging and social withdrawal in the progeny.^84^ Unfortunately, as Vit.D cannot be used in human pregnancy because of its detrimental hypercalcaemic effects on the foetus,^85^ postnatal studies have been performed with the safer form cholecalciferol (*vide infra*). Finally, oral probiotics given to experimental MIA dams prevent offspring from developing stereotypical behaviours, social withdrawal and reduction in cortical parvalbumin–GABA neurons.^86^

### Postnatal intervention in humans

The use of corticosteroids or immunosuppressive agents achieves the amelioration of expressive language disorder, social withdrawal and behavioural disruption in single cases or brief case series.^87^ Supplementation with the cyclooxygenase‐2‐non‐steroidal celecoxib and, more recently, with prednisolone as add‐on to D2‐blocker risperidone on randomised, controlled trials has remarkably improved core clinical features in children with a regressive form of autism.^88^ Mesenchymal stem cell transplantation, known to have a strong immune regulatory potential, has also significantly improved behavioural disorders in some children with ASD.^88^ On the contrary, intravenous immunoglobulin has been inconclusively tested as immunotherapy for autism. This result speaks against a peripherally maintained B‐cell immune process and suggests that we need more careful pathophysiological studies.^89^

Dietary intervention with Vit.D is based on a supposed, but not always confirmed,^79^, ^90^ Vit.D deficiency in the serum of children with ASD, and on the fact that the level of Vit.D is inversely correlated with language and behavioural scores on Childhood Autism Rating Scale.^91^ Vit.D has immunomodulatory and neuroprotective functions, particularly during pregnancy; its serum shortfall may therefore contribute to the persistence of both immune and behavioural abnormalities in post‐gestational age. Vit.D’s biological activity depends on the Vit.D receptor (VDR) gene polymorphisms. We evaluated VDR polymorphic allele distribution in a large cohort of families with autistic children and found that the Vit.D/VDR complex with low biological activity is prevalent in children with ASD and their mothers.^92^ Our findings encourage Vit.D supplementation in preventative and therapeutic protocols for ASD. Conformingly, dietary Vit.D supplementation in children with ASD seems to improve significantly their behavioural outcomes and, concomitantly, to reduce the CD5 T‐cell blood level.^93^

## Discrepancies in the (auto)immune hypothesis of ASD

A footprint of the immune system is evident in every biological process, including typical neurodevelopment. Historically, several immune proteins, including cytokines, have been conventionally divided into pro‐ or anti‐inflammatory functional classes and considered able of skewing the immune system towards a good or a bad response. Against this traditional view, modern neuroimmunology indicates that immune effectors, also produced by glial cells and neural precursors,^94^ can have two possible antithetical roles, as they are either beneficial or detrimental depending on several factors: homeostasis of the neural tissue, duration of the stressor, neurodevelopmental stage (e.g. embryonic, foetal, early postnatal) and other influencing variables (inherited or *de novo‐*mutated genes, epigenome, environment and gender).

On the basis of the above‐described ASD risk factors such as familial autoimmunity, common non‐neurological autoimmune comorbidities and gestational immune activation because of an active maternal autoimmune disease, several studies put forward the aetiological hypothesis of a classical autoimmune environmental–genetic interaction for ASD.^95^ Thus, the adaptive immune branch has been extensively studied, although, after the strong initial claim, the evidence that autism may be an intrinsically autoimmune disorder seems not to have stood the test of time.

In contrast to most autoimmune diseases, the gender ratio of affected individuals with ASD is exceedingly in favor of male individuals.^2^ Despite the fact that autoantibodies to brain antigens have been repeatedly described both in children with ASD and their mothers,^70^, ^96^ the level of evidence is low, and we lack validation by independent research groups and the specificities of maternal and offspring antibodies. A recent systematic review suggests that there is currently insufficient evidence to develop a guideline for routine autoantibody testing in ASD patients.^71^ As for other supposed biomarkers of adaptive immunity, recent profilings in sporadic cases and in ASD discordant sibling pairs do not confirm the initial claim of specific, Th1‐skewed profiling in ASD.^78^, ^79^ Also, dysregulation of the adaptive T‐cell immunity seems not to associate with clinical severity, head circumference, gastrointestinal or allergic issues of children with ASD and the only correlation is found between the level of innate cytokines IL‐1α/β and quantitative traits at the Vineland Scale.^78^

The limits of studies on peripheral blood markers can also be attributed to between‐study heterogeneity, to small sample sizes, to techniques with different sensitivity and specificity and to epiphenomenal factors such as the psychological distress of reluctant children with autism before a venipuncture. Another critical issue is that immune parameters measured *in vitro* in the periphery can be misleading with regard to the relevant regulation *in vivo* and in the central nervous system. Nonetheless, fragmentary immune findings are said to be associated with a peculiar onset type of autism or with symptom severity. These findings, however, only account for a part of the disorder and not for the whole clinical picture.

Data on the immune dysregulation in ASD are beginning to be consolidated, but given its clinical heterogeneity, it remains difficult to explain different behavioural outcomes based solely on immune phenotype. Therefore, to avoid spurious conclusions, it would be important to aim at well‐defined and homogeneous experimental populations with homogeneous ASD core symptoms.

Many of the reviewed results have been obtained in the pre‐modern neuroimmunology era, a time when neuroscientific approaches aimed at including ASD among the prototypical antibody‐ or cell‐mediated diseases, and when the 'immunological brain privilege' was believed to prevent the crosstalk between the brain and the immune system. This long‐standing dogma, recently disproven by the discovery of glymphatic and meningeal lymphatic vessels,^97^ is a misconception that slowed down scientific progress in neurodevelopmental sciences and contributed to spurious conclusions. Thanks to the fruitful MIA models, research in autism has recently evolved and now involves a less 'disease‐oriented' analysis, focusing instead on how the maternal–foetal interface really works, how the immune system impacts a developing brain during pregnancy, how foetal neuroglia cells play an immune regulatory role, and how this can affect the susceptibility to neurological, behavioural and mood disorders in neonates, toddlers and adolescents.

### Perspectives

Our immunological understanding of ASD aetiology is still at the beginning, and this review probably asks more questions than it answers. The traditional view of 'one disease–one individual' is changing in favor of a new 'multifaceted' paradigm, since the prenatal environment is shared by two half‐different individuals, the mother and the child. The maternal–foetal interface appears to be the most vulnerable site for the appearance of neurodevelopmental disorders, with gestational events contributing to atypical microglial activation, neurogenesis, migration, differentiation, synapse formation and pruning. Neuroglial function is still under evaluation in ASD, but it is likely crucial in maintaining, and perhaps initiating, some of the brain abnormalities observed in autism: there is evidence that complement C1q is expressed by synapses of perinatal (and not adult) neurons.^40^

New studies also bring new ideas on how emerging microbes such as Zika^98^ or gut microbiome^15^ can influence brain shaping during development and predispose to lifelong immune‐mediated neurological and psychiatric diseases. The application of genetic panels and new genotype–phenotype mapping^99^ will diversify patient populations and enhance our understanding of the cryptic genetic composition of ASD. The interface generated by merging next‐generation sequencing, modern neuroimmunology and neuroimaging advancements will help developing a more personalised treatment for autistic children.

Nonetheless, pregnancy is also a time in which interventions may be most effective in preventing ASD. Preventative strategies include treatment of prolonged fevers, maternal diet and correction of possible epigenetic forces such as maternal obesity, diabetes and hypertension.^95^ Millions of pregnant women are exposed to infections each year. Thus, reducing exposure to infections, controlling risk factors and implementing an appropriate management of the maternal immune response could have a significant impact on public health.^100^

## Conflict of interest

The authors declare no conflict of interest.

## Authors’ Contributions

All authors participated in the writing of the manuscript and were involved in drafting the manuscript or revising it critically for important intellectual content. Each author has participated sufficiently to take public responsibility for appropriate portions of the content. All gave final approval of the submitted version.

## Ethical approval

The article was written in accordance with the Code of Ethics of the World Medical Association (Declaration of Helsinki) for experiments involving humans. The manuscript is in line with the Recommendations for the Conduct, Reporting, Editing and Publication of Scholarly Work in Medical Journals and aims for the inclusion of representative human populations (sex, age and ethnicity) as per those recommendations.
